# Factors Related to Significant Improvement of Estimated Glomerular Filtration Rates in Chronic Hepatitis B Patients Receiving Telbivudine Therapy

**DOI:** 10.1155/2017/4192169

**Published:** 2017-07-05

**Authors:** Te-Fu Lin, Ping-I Hsu, Kung-Hung Lin, Feng-Woei Tsay, Tzung-Jiun Tsai, Yan-Hua Chen, Hsien-Chung Yu

**Affiliations:** ^1^Division of Gastroenterology and Hepatology, Department of Internal Medicine, Kaohsiung Veterans General Hospital, Kaohsiung, Taiwan; ^2^National Yang-Ming University, Taipei, Taiwan; ^3^Department of Nursing, Meiho University, Pingtung, Taiwan

## Abstract

**Background and Aim:**

The improvement of estimated glomerular filtration rates (eGFRs) in chronic hepatitis B (CHB) patients receiving telbivudine therapy is well known. The aim of this study was to clarify the kinetics of eGFRs and to identify the significant factors related to the improvement of eGFRs in telbivudine-treated CHB patients in a real-world setting.

**Methods:**

Serial eGFRs were calculated every 3 months using the Chronic Kidney Disease Epidemiology Collaboration (CKD-EPI) equation. The patients were classified as CKD-1, -2, or -3 according to a baseline eGFR of ≥90, 60–89, or <60 mL/min/1.73 m^2^, respectively. A significant improvement of eGFR was defined as a more than 10% increase from the baseline.

**Results:**

A total of 129 patients were enrolled, of whom 36% had significantly improved eGFRs. According to a multivariate analysis, diabetes mellitus (DM) (*p* = 0.028) and CKD-3 (*p* = 0.043) were both significantly related to such improvement. The rates of significant improvement of eGFR were about 73% and 77% in patients with DM and CKD-3, respectively.

**Conclusions:**

Telbivudine is an alternative drug of choice for the treatment of hepatitis B patients for whom renal safety is a concern, especially patients with DM and CKD-3.

## 1. Introduction

The popularity of oral nucleos(t)ide analogues (NAs) for the treatment of chronic hepatitis B (CHB) has increased substantially for years in a real-world setting [1, 2]. Renal toxicity is a matter of concern, however, when the use of NAs occurs over a prolonged period because the clearance of all NAs must occur via the kidneys [3–5]. Numerous studies have shown that tenofovir or adefovir dipivoxil exposure is associated with significant declines in the estimated glomerular filtration rate (eGFR) in CHB patients [6–9]. Mild declines in eGFR have been also documented in treatment with all kinds of NAs except for telbivudine [4, 10–12]. Rather, past studies have actually reported the improvement of eGFRs with telbivudine treatment [13, 14]. In addition, telbivudine therapy has also been documented to have the effect of improved eGFRs in decompensated, cirrhotic CHB patients [15]. Durable effects were also noted in the results of a 4-year extended study of telbivudine-treated CHB patients who had no drug resistance during the initial 2-year treatment [16]. In addition, old age (i.e., an age of more than 50 years) and mild renal function impairment (i.e., an eGFR of 60–90 mL/min/1.73 m^2^) have been found to be associated with greater eGFR improvement in patients receiving telbivudine treatment [13]. Some real-world data have also confirmed these findings [14, 17]. However, the mechanism for the increase in eGFR in telbivudine therapy remains unclear.

To date, insufficient attention has been given to the renal safety of telbivudine in real-world practice, partly because it is no longer recommended as a first-line therapy due to the higher drug resistance of patients to it than to entecavir or tenofovir [18–20]. Nevertheless, in some Asian countries where hepatitis B is endemic and treatment options are limited, telbivudine remains the drug most widely available for the treatment of CHB due to its good efficacy, good tolerability, and relatively low cost [21–23]. Thus, the following question must be asked: What kind of patients will benefit from telbivudine therapy with no compromise of renal safety and minimal drug resistance? As is well known, the roadmap approach will significantly decrease the risk of drug resistance in clinical practice in telbivudine-treated patients [23–29]. However, there is insufficient data regarding the renal protective effects of telbivudine therapy for special populations in real-world contexts. Relatedly, it seems important that renal safety be considered when choosing telbivudine for the treatment of CHB patients.

Consequently, we performed the current retrospective analysis, which was based on the data of our cohort studies of CHB patients undergoing telbivudine therapy. We aimed to clarify the individual changes in eGFRs over time and the differences in eGFRs between subgroups in a real-world setting. In addition, the factors related to the improvement of eGFRs were also determined. To assess renal function more accurately in the normal range, we used the Chronic Kidney Disease Epidemiology Collaboration (CKD-EPI) equation to calculate eGFRs [30]. This formula is well established and more accurate in patients with normal or slightly impaired renal function, function levels that are consistent with those of the patient population we see in our clinical practice.

## 2. Methods

We retrospectively enrolled consecutive CHB patients who were treated with telbivudine (600 mg once daily) at Kaohsiung Veterans General Hospital from 2008 to 2010 in our two previous cohort studies, which were designed with two purposes: (1) to verify the roadmap model through adefovir add-on therapy at month 6 and (2) to investigate the role of genotype and the kinetics of quantitative hepatitis B surface antigen on the efficacy of telbivudine therapy [27]. These two previous studies were approved by the Ethics Committee and the Institutional Review Board of the Kaohsiung Veterans General Hospital, and all the patients gave their written informed consent.

All of the patients fulfilled the following criteria at the initiation of treatment: (1) positive for HBsAg for more than 6 months; (2) ALT level of at least twice the upper limit of normal; (3) hepatitis B virus (HBV) DNA levels > 20,000 IU/mL in hepatitis B e-antigen (HBeAg)-positive patients, HBV DNA levels > 2000 IU/mL in HBeAg-negative patients, or hepatic decompensation (total bilirubin level > 2 mg/dL or prolongation of prothrombin time by >3 seconds) with detectable HBV DNA irrespective of the level; (4) no coinfection with hepatitis C virus and human immunodeficiency virus; and (5) treatment-naïve patients or lamivudine-experienced patients who did not develop genotypic resistance to lamivudine. Patients who received telbivudine therapy for more than 24 months without concomitant usage of nephrotoxic drugs other than NAs or acute kidney injury during treatment from other etiologies were enrolled for further analysis in the present study. Patients who developed telbivudine-related myopathy or neuropathy and had been switched to other NA (entecavir or tenofovir) were also excluded in the present study.

The baseline characteristics, serologic markers, HBV DNA levels, HBV genotypes, alpha-fetoprotein levels, comorbidities, and liver disease status of each patient were recorded, while the genotypic resistance to lamivudine was checked before the initiation of telbivudine therapy in lamivudine-experienced patients. The patients attended special clinics for regular follow-up visits. The serologic markers, hematological and biochemical parameters, and HBV DNA levels of the patients were assessed every 3 months. Serum creatinine levels were assessed every 3 months for safety reasons in our initial study design. The eGFR of each patient was calculated using the CKD-EPI equation [30].

Some patients without a complete virological response (HBV DNA level < 60 IU/mL) at month 6 received adefovir (10 mg once daily) add-on therapy according to the roadmap concept [25, 27]. Some patients with virological breakthrough (defined as an increase in HBV DNA levels by more than 1 log_10_ IU/mL above the nadir during treatment) during telbivudine monotherapy also received adefovir add-on therapy as a rescue treatment. All the patients were prospectively followed up for 24 months from the initiation of telbivudine therapy.

HBV DNA levels were measured using Abbott RealTime HBV assays (Abbott Molecular Inc., Des Plaines, IL, USA) with a lower detection limit of 10 IU/mL. HBV genotype and genotypic mutations were determined by direct DNA sequencing (SeqHepB; Abbott Diagnostics, Lake Forest, IL, USA). Serum HBsAg, HBeAg, and anti-HBe antibodies were measured using radioimmunoassay kits (Ausria II-125; Abbott Laboratories, North Chicago, IL, USA). Hematological and biochemical parameters, including the serum creatinine level, were measured using automatic analyzers in a central laboratory in our hospital.

Patients were classified as CKD-1, -2, or -3 according to a baseline eGFR of ≥90, 60–89, or <60 mL/min/1.73 m^2^, respectively [31]. According to the 4-year extended GLOBE study, lamivudine therapy also achieved a mean 8.9% increase in eGFRs from baseline values after administration of the initial therapy for 2 years [16]. Therefore, in the present study, we defined the improvement, maintenance, and deterioration of eGFR by, respectively, an increase in eGFR > 10%, an increase or decrease in eGFR of ≤10%, and a decrease in eGFR > 10% from the baseline over a period of 24 months.

All statistical analyses were performed using STATA version 10 (STATA Corp, College Station, TX, USA). Pearson *χ*^2^ analysis or Fisher's exact test was used for the comparison of categorical variables, while continuous variables were compared using the Student *t*-test or the Mann–Whitney *U* test where appropriate. For the correlation of each pair of observations in our study was different, a generalized estimating equation (GEE) was used to compare the individual changes in eGFRs over time. Bonferroni correction and the least significant difference test were used to compare the differences in the changes in the mean eGFRs of the subgroups and for different time intervals. The McNemar-Bowker test was used to check the significance of table indicating the altered CKD statuses of eGFR from baseline to month 12 and month 24. Logistic regression models were used to estimate the factors related to increases of more than 10% in the eGFR at month 24. Variables with marginal statistical significance (*p* < 0.1) in the univariate analysis were subjected to multivariate analysis. A two-tailed *p* value of <0.05 was considered significant in all tests.

## 3. Results

**Figure 1:**
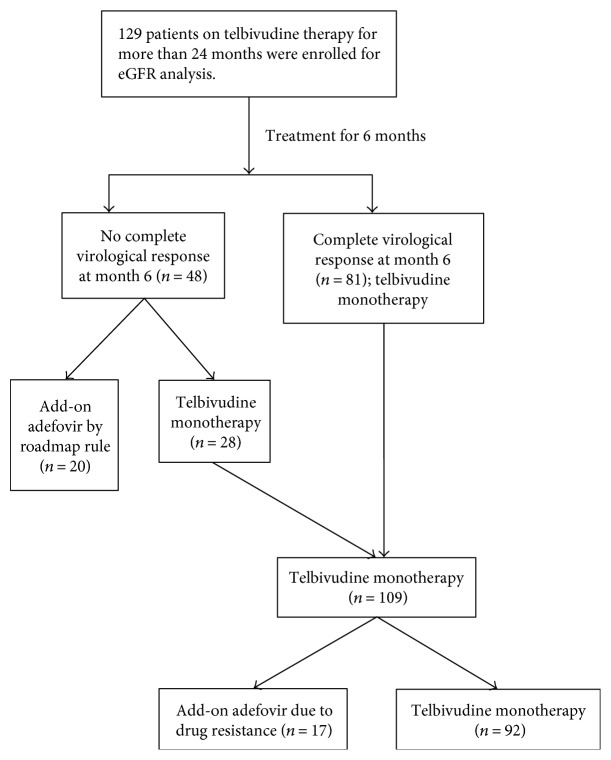
The flow chart of treatment of enrolled patients according to the roadmap rule.

A total of 129 patients who received telbivudine therapy for more than 24 months were enrolled. The clinical characteristics of the patients are summarized in Table 1. One hundred and twelve patients were treatment-naïve, and 17 patients were lamivudine-experienced. As shown in Figure 1, 81 of the patients (63%) achieved a complete virological response at month 6. Of the remaining patients (*n* = 48), 20 patients received early add-on adefovir therapy at month 6 according to the roadmap rule and 28 patients requested continued telbivudine monotherapy. Hence, 101 of the patients (78%) obeyed the roadmap rule in our practice. Of the patients (*n* = 20) who received the early add-on adefovir therapy, no patients developed virological breakthrough or genotypic resistance within 24 months. In contrast, of the 109 patients who received telbivudine monotherapy, 17 patients developed genotypic resistance to telbivudine and received salvage adefovir add-on therapy at different time points within 24 months. The rate of genotypic resistance was significantly lower (3 of 101 patients) in the patients treated according to the roadmap rule than in the other patients (14 of 28 patients) (3% versus 50%; *p* < 0.01). In addition, the rate of genotypic resistance was also lower in the treatment-naïve patients (12 of 112 patients) than in the lamivudine-experienced patients (5 of 17 patients) (11% versus 29%; *p* = 0.137).

**Figure 2:**
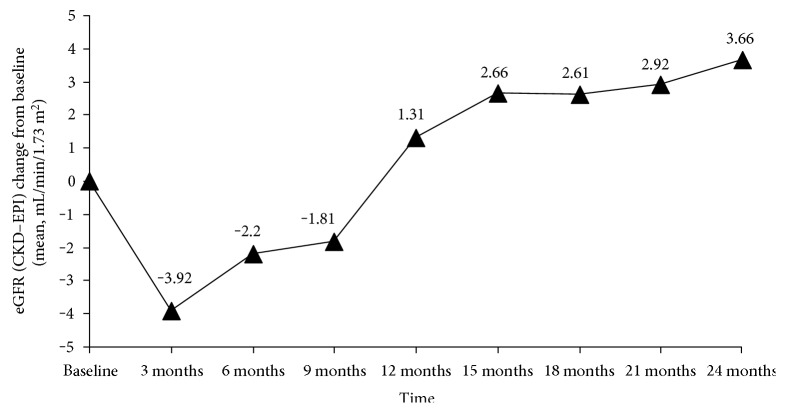
The changes of eGFR from baseline over time in all the patients (*n* = 129). A transient decline at month 3 followed by a gradual increase thereafter is shown, with statistical significance after month 12 (*p* < 0.01).

The changes in the mean eGFR from the baseline to subsequent time points are shown in Figure 2. In general, the kinetics of the eGFR changes were as follows: a transient decline at month 3 followed by a gradual recovery between month 3 and month 9 and then a significant increase from month 12 to month 24 (*p* < 0.01). A subgroup analysis revealed that the kinetics of the eGFR were similar irrespective of the status of cirrhosis, decompensation, HBeAg, baseline HBV DNA, and the virological response at month 6 (all *p* > 0.05 in comparisons of status; all *p* < 0.05 in comparisons of time intervals) (Table 2). However, different eGFR kinetics were noted for patients of different genotypes (*p* = 0.02) and ages (*p* = 0.01). In genotype C patients, the transient decline in the eGFR at month 3 disappeared (*p* = 1.0, by least significant difference test). Nevertheless, the pattern of increase in the eGFR was similar in both genotypes from month 12 (*p* = 0.116). On the other hand, in patients with age ≥ 50 years, the increase in the eGFR was less significant than in patients with age < 50 years (*p* = 0.004) (Table 2).

A total of 37 patients received adefovir add-on therapy at different time points during the telbivudine treatment. Twenty-nine patients who received add-on adefovir therapy for more than 12 months were enrolled for further analysis to evaluate the influence of adefovir add-on therapy on the kinetics of eGFR. The increase in the eGFR (mean; +6.51 mL/min/1.73 m^2^) after adefovir add-on therapy was still noted over time but did not achieve statistical significance (*p* = 0.293). For the remaining 109 patients who receiving telbivudine monotherapy, improvement of eGFR was still significant (*p* < 0.01). Hence, the effect of improvement of eGFR by telbivudine seemingly became weaker in adefovir add-on patients.

Significant increases in the eGFR were noted in patients with CKD-2 from month 15 (*p* = 0.007) and in patients with CKD-3 from month 24 (*p* = 0.004) but not in patients with CKD-1 (*p* > 0.1). The table indicating the altered CKD statuses of eGFR for different CKD groups at month 24 is presented in Table 3. At month 24, 85% and 33% of the patients with CKD-3 and CKD-2 were switched to CKD-2 and CKD-1 (*p* = 0.006), respectively. In contrast, 2% and 25% of patients with CKD-2 and CKD-1 were switched to CKD-3 and CKD-2.

At month 24, the rates of improvement, maintenance, and deterioration of eGFR were 36% (*n* = 47), 48% (*n* = 61), and 16% (*n* = 21), respectively. A multivariate analysis revealed that diabetes mellitus (DM) (hazard ratio, 4.19; 95% C.I., 1.17–15.10; *p* = 0.028) and CKD-3 (hazard ratio, 4.56; 95% C.I., 1.05–19.88; *p* = 0.043) were two factors related to the improvement of eGFR (Table 4). In contrast, there was no significant factor related to the significant deterioration of eGFR by multivariate analysis (not shown).

**Figure 3:**
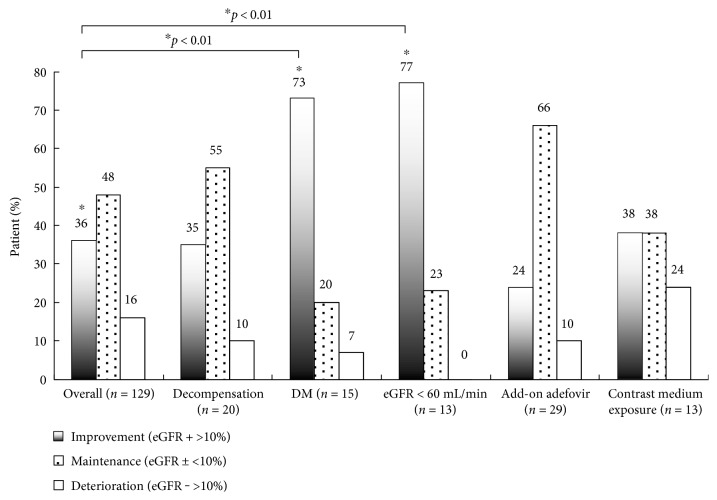
The rates of improvement, maintenance, and deterioration of eGFR in all patients and in patients at high risk for renal toxicity, including hepatic decompensation (*n* = 20), DM (*n* = 15), CKD-3 (*n* = 13), concomitant usage of adefovir (*n* = 29), and contrast medium exposure (*n* = 13). The rates of improvement of eGFR in patients with DM and CKD-3 were significantly higher than the rate for all the patients (both *p* < 0.01).

The rates of improvement, maintenance, and deterioration of eGFR in patients with so-called high risk for renal toxicity [18], including those with hepatic decompensation, DM, CKD-3, concomitant usage of adefovir, and contrast medium exposure, are shown in Figure 3. The rates of significant improvement of eGFR in patients with DM (73%) and CKD-3 (77%) were higher than the rate in all the patients (both *p* < 0.01). On the other hand, about 24% of patients with contrast medium exposure exhibited eGFR deterioration over the 24 months. However, the rate of deterioration of eGFR in these patients was not statistically significant (*p* > 0.1).

## 4. Discussion

In the current study, we investigated the kinetics of the eGFR in CHB patients treated with telbivudine therapy. The results clearly demonstrated a significant increase in the eGFR after treatment with telbivudine for more than 12 months. In addition, the patterns of eGFR increase were similar in various subgroups irrespective of the status of liver disease, viral factors, and the treatment response. These results are comparable with the findings of other studies [13–17]. Moreover, the improvement of eGFR levels was also significant in patients with high renal risk, especially in the patients with DM and CKD-3. Even in patients with CKD-3 that was considered to be irreversible, the majority (77%) of patients achieved significant improvement of the eGFR over 24 months. In a previous prospective study of telbivudine-treated HBeAg-positive patients, the increase in the eGFR levels was most significant in patients with eGFRs < 60 mL/min, compared to patients with eGFRs of 60–80 and >80 mL/min [29]. Therefore, this study validates the renal protective effect of telbivudine therapy in terms of the eGFR in CHB patients, particularly in patients with baseline renal insufficiency. Hence, for CHB patients with high renal risk, especially those with DM and CKD-3, telbivudine should be one of the primary choices for antiviral therapy [17, 32]. Nevertheless, due to no control group with other NAs in our study, it needs further investigation whether other NAs also achieve a significant increase in eGFR in CHB patients with DM and CKD-3.

The mechanism of renal protection of telbivudine therapy in terms of the eGFR remains unclear. Chan et al. suggested that possible mechanisms for such improvement include increased renal blood flow and decreased tubular dysfunction [15]. Liang et al. reported that telbivudine could decrease serum angiotensin converting enzyme (ACE) levels, blocking the renin-angiotensin aldosterone regulatory system and inhibiting systemic vasoconstriction, renal sodium, and renal fluid retention [33]. Moreover, noninsulin-dependent diabetic patients associated with nephropathy had significantly higher plasma angiotensin II levels compared with noncomplicated diabetics [34]. Since ACE plays an important role in the processing of angiotensin I to angiotensin II, reduced ACE levels are associated with lower angiotension II levels. This may provide a possible explanation of why DM is a significant factor in the significant improvement of eGFR via telbivudine treatment. However, further investigations are needed to clarify this pharmaco-pathophysiology.

Adefovir add-on therapy was the choice of salvage treatment in the current study. A recent long-term investigation demonstrated a significant decline of renal function in patients with adefovir add-on lamivudine therapy used as a rescue treatment [35]. Doubtless, the renal toxicity of adefovir was the leading cause of the said decline [3–7]. In contrast to lamivudine, however, telbivudine therapy has been reported by some studies to have a renal protective effect even with add-on adefovir therapy [36, 37]. In our study, the kinetics of eGFR in patients with adefovir add-on therapy revealed only an insignificant increase, with the renal protective effect in eGFR seemingly becoming weaker in this group. The discrepant results might be due to the small case series in our study. However, no patient experienced a decline in the eGFR > 20% from the baseline with concomitant usage of telbivudine and adefovir.

In fact, it has been reported that treatment of hepatitis B with NAs can improve renal function in CHB patients with underlying HBV-related renal disease [38]. Because of the retrospective nature of our study, however, we did not check the key factors to evaluate the possibility of HBV-related or other etiologies related to renal diseases in our patients. Mallet et al. reported that patients who were born in HBV endemic areas with initial high HBV DNA levels (more than 5 log IU/mL) were more likely to have increased eGFR with treatment by NAs other than telbivudine [11]. However, the renal protective effect of telbivudine therapy in terms of improved eGFRs was found to be unique, durable, and unrelated to baseline HBV DNA levels. In the GLOBE study of extensional therapy consisting of a switch from lamivudine to telbivudine, an additional increase in eGFRs was noted after switching to telbivudine for 2 additional years [16].

According to the guidelines [18], the major populations with high renal risk were mostly taken into consideration in our study. Indeed, there are still some possible factors affecting eGFR, such as usage of nephrotoxic drugs and body mass index. We had excluded the patients receiving long-term nephrotoxic drugs initially. In addition, the body mass index was not considered for analysis due to fluctuation of data during treatment and unreliable in cirrhotic patients with ascites. Moreover, in other studies about the NAs on the renal function, body mass index was not considered to be a major factor [10–12]. Thus, we thought that these possible factors would be minor factors. On the other hand, in our study, 16% of patients of CHB taking telbivudine had eGFR deterioration after 24 months treatment. Similar to our findings, about 12% eGFR deterioration in terms of CKD stage was also noted in a recent study [17]. Indeed, some patients experienced deterioration of eGFR during telbivudine treatment. To our knowledge, the reason for the deterioration of eGFR should be complex. However, we did not find significant factor related to the significant deterioration of eGFR by multivariate analysis. Hence, further studies are needed to clarify this issue.

There are some limitations to this study. First, this was a retrospective analysis and we did not check the parameters for the possibility of baseline HBV or other etiology-related renal diseases in our patients. Second, we did not investigate the etiology of the early eGFR decline and consequent eGFR increases; detailed studies may be needed for further investigation of these phenomena. However, this study reflects a real-world scenario and demonstrates the renal protective effect of telbivudine therapy in terms of the eGFR under routine clinical conditions. Finally, an increase in the eGFR may not truly reflect improved renal function. Especially in DM patients, the related hyperperfusion reflects the early damage to renal function before the development of proteinuria. In this study, we had insufficient data regarding the baseline renal status of our DM patients. Further studies are thus needed to clarify this issue.

In conclusion, significant improvement of the eGFR was achieved in 36% of the CHB patients treated with telbivudine therapy over 24 months. The renal protective effect of the telbivudine therapy in terms of the eGFR was unique irrespective of the status of variant virological, hepatic factors, the concomitant usage of adefovir, and DM comorbidity. In addition, significant improvements in the eGFR were noted, especially in patients with CKD-3 and DM. For these kinds of patients, in addition to adhering to the roadmap rule, clinicians should consider telbivudine as an alternative drug of choice due to concerns regarding renal safety.

## Figures and Tables

**Table 1:** Baseline characteristics of the chronic hepatitis B patients (*N* = 129) treated with telbivudine therapy.

Characteristic	Patients *n* (%)
Age, years (mean ± SD)	48 ± 13
Gender, male	99 (77)
HBeAg status, positive	46 (36)
Genotype, B/C/unknown	73/23/33
Treatment-naïve	112 (87)
Liver cirrhosis	31 (24)
Hepatic decompensation	20 (16)
Hepatocellular carcinoma	13 (10)
Diabetes mellitus	15 (12)
Hypertension	13 (10)
Contrast-medium exposure	13 (10)
Albumin, g/dL (IQR)	4.3 (4.0–4.5)
Bilirubin, mg/dL (IQR)	0.9 (0.7–1.3)
AST, U/L (IQR)	80 (51–162)
ALT, U/L (IQR)	158 (85–337)
Creatinine (mg/dL) (mean ± SD)	1.06 ± 0.59
CKD-EPI (mL/min/1.73 m^2^) (mean ± SD)	85.04 ± 18.27
Baseline HBV DNA, log IU/mL (IQR)	5.9 (4.8–7.5)
Baseline HBV DNA > 7 log IU/mL	42 (33)

ALT: alanine transaminase; AST: aspartate transaminase; CKD-EPI: the formula of Chronic Kidney Disease Epidemiology Collaboration; HBeAg: hepatitis B e-antigen; IQR: interquartile range; SD: standard deviation.

**Table 2:** Comparisons of the changes in the mean eGFR (CKD-EPI) of different subgroups of chronic hepatitis B patients treated with telbivudine therapy over time.

		Wald chi-square	df	*p* value
HBeAg	Positive versus negative	1.04	1	0.308
	Time	31.21	7	0.000
	Interaction term	14.32	7	0.046
Cirrhosis	Yes versus no	0.16	1	0.691
	Time	24.57	7	0.001
	Interaction term	1.34	7	0.987
Complete response at 6th month	Yes versus no	0.26	1	0.607
	Time	34.30	7	0.000
	Interaction term	7.81	7	0.350
Decompensation	Yes versus no	2.01	1	0.156
	Time	21.66	7	0.003
	Interaction term	5.37	7	0.614
HBV DNA ≥ 7 log IU/mL	Yes versus no	0.00	1	0.966
	Time	32.72	7	0.000
	Interaction term	6.40	7	0.494
Age ≥ 50 years	Yes versus no	11.77	1	0.001
	Time	37.42	7	0.000
	Interaction term	21.15	7	0.004
Genotype	B versus C	5.39	1	0.020
	Time	19.14	7	0.008
	Interaction term	11.57	7	0.116

Analysis by generalized estimating equations. df: degree of freedom.

**Table 3:** Table indicating the altered CKD statuses of eGFR (CKD-EPI) changes at month 12 and month 24 according to the baseline eGFR (CKD-EPI) levels.

	eGFR (mL/min/1.73 m^2^)	12 months, *n* (%)			Total	*p* value
		<60	60–89	≥90		
Baseline *n* (%)	<60	5 (38)	8 (62)	0 (0)	13 (100)	0.057
	60–89	1 (2)	46 (72)	17 (26)	64 (100)	
	≥90	0 (0)	14 (27)	38 (73)	52 (100)	
Total		6 (5)	68 (52)	55 (43)	129 (100)	

	eGFR (mL/min/1.73 m^2^)	24 months, *n* (%)			Total	*p* value
		<60	60–89	≥90		

Baseline *n* (%)	<60	2 (15)	11 (85)	0 (0)	13 (100)	0.006
	60–89	1 (2)	42 (65)	21 (33)	64 (100)	
	≥90	0 (0)	13 (25)	39 (75)	52 (100)	
Total		3 (2)	66 (51)	60 (47)	129 (100)	

**Table 4:** Factors related to the improvement of eGFR (CKD-EPI) (increase in eGFR > 10%) according to univariate and multivariate logistic regressions.

Risk factor	Univariate	*p* value	Multivariate	*p* value
	HR (95% CI)		HR (95%CI)	
Age: ≥50 years	2.221 (1.070–4.611)	0.032	1.315 (0.576–3.001)	0.516
Sex: male	0.987 (0.423–2.305)	0.976		
HBeAg: positive	0.663 (0.308–1.427)	0.293		
Cirrhosis	0.785 (0.333–1.849)	0.580		
Decompensation	0.929 (0.342–2.520)	0.885		
Hepatocellular carcinoma	1.101 (0.338–3.583)	0.873		
HBV genotype: type B	0.518 (0.222–1.209)	0.128		
Baseline ALT > 200 U/L	1.031 (0.494–2.154)	0.935		
Baseline HBV DNA: <7 log IU/mL	1.222 (0.564–2.650)	0.611		
Baseline eGFR < 60 mL/min/1.73 m^2^	7.117 (1.849–27.399)	0.004	4.560 (1.046–19.875)	0.043
Diabetes mellitus	5.958 (1.776–19.993)	0.004	4.194 (1.165–15.098)	0.028
Hypertension	0.931 (0.436–3.874)	0.864		
Add-on adefovir therapy	0.477 (0.186–1.222)	0.123		
Complete virological response at month 6	1.670 (0.778–3.585)	0.189		
Contrast medium exposure	1.101 (0.338–3.583)	0.873		

ALT: alanine transaminase; HBeAg: hepatitis B e-antigen; HR: hazard ratio.
